# Chronic Thromboembolic Pulmonary Hypertension in a Child With Sickle Cell Disease

**DOI:** 10.3389/fped.2020.00363

**Published:** 2020-07-24

**Authors:** Robert Spencer, Gerson Valencia Villeda, Koji Takeda, Erika B. Rosenzweig

**Affiliations:** ^1^Division of Pediatric Cardiology, Morgan Stanley Children's Hospital, New York, NY, United States; ^2^Division of Pediatric Cardiology, Arnold Palmer Hospital for Children, Orlando, FL, United States; ^3^Division of Cardiothoracic Surgery, Columbia University Medical Center, New York, NY, United States

**Keywords:** pediatric cardiology, pulmonary hypertension, CTEPH—chronic thromboembolic pulmonary hypertension, sickle cell disease, hematology

## Abstract

Chronic thromboembolic pulmonary hypertension is a potentially curable form of pre-capillary pulmonary hypertension (PH) resulting from incomplete resolution of pulmonary thromboemboli. We describe an 11-year-old boy with homozygous sickle cell disease with an indwelling catheter found to have severe PH on routine screening echocardiography. The diagnosis was confirmed by CT, ventilation-perfusion scintigraphy, and right heart catheterization. The patient was medically managed until undergoing pulmonary thromboendarterectomy with resolution of his PH. This case highlights the need for pediatric providers to be aware of this underdiagnosed form of PH, particularly for patients at high risk.

## Introduction

Chronic thromboembolic pulmonary hypertension (CTEPH) is a distinct form of pulmonary hypertension (PH) that results from unresolved acute pulmonary embolism. The disease is caused by mechanical obstruction of the pulmonary arteries by chronic, fibrotic organized thrombi (1, 2). It is rarely diagnosed in children and has an unknown incidence in the general population (3–5).

Early diagnosis and treatment are critical, particularly because patients will develop severe PH and right heart failure. When recognized in a timely manner, the disease is often curable by pulmonary thromboendarterectomy (PTE) (6–9). We report an 11-year-old boy with sickle cell disease and an indwelling venous catheter found to have elevated right ventricular (RV) systolic pressure on routine echocardiography. Further workup led to the diagnosis of CTEPH, and he was successfully treated with PTE.

## Case

An 11-year-old boy with hemoglobin SS sickle cell disease (SCD) was referred to our hospital for further treatment of CTEPH. He had a history of multiple pain crises, acute chest syndrome, and acute ischemic strokes at ages 3 and 7 years. His SCD was further complicated by moyamoya syndrome, for which he underwent encephalodurosynangiosis at age 7 years. His hypercoagulability workup had been negative for antithrombin deficiency, protein C deficiency, protein S deficiency, factor V Leiden, plasminogen deficiency, and anticardiolipin antibodies.

Approximately 10 months before his referral, routine screening transthoracic echocardiogram (TTE) revealed a right atrial (RA) thrombus thought to be related to his Broviac central venous catheter, which had been used for exchange transfusions. He was started on enoxaparin and his Broviac catheter was replaced. TTEs over the ensuing months demonstrated persistent RA thrombus without change in size, with normal RV pressure and normal biventricular systolic function.

Seven months later, the patient was electively admitted to the referring institution in anticipation of a bone marrow transplant (BMT), at which time routine TTE showed an elevated RV systolic pressure (58 mmHg plus the RA pressure), a change from his previous echocardiograms. CT scan of the chest at that time revealed multiple bilateral lower lobe and left upper lobe pulmonary emboli. Clinically, he reported mild dyspnea on exertion and exercise intolerance for several weeks. Medical management was started with bosentan and the patient's enoxaparin dose was increased.

Follow-up TTE performed 3 months later showed severe PH, with RV systolic pressure of 90 mmHg plus the RA pressure and a corresponding blood pressure of 108/52 mmHg. This raised suspicion for CTEPH, which was supported by a ventilation-perfusion (VQ) scan showing multiple areas of wedge-shaped mismatched perfusion defects consistent with chronic bilateral thromboembolic disease and secondary PH consistent with CTEPH. The following day, right heart catheterization demonstrated pulmonary arterial pressure of 80/35 mmHg with a mean of 52 mmHg, pulmonary capillary wedge pressure of 14 mmHg, cardiac index of 5.7 L/min/m^2^, and pulmonary vascular resistance index of 6.6 WUm^2^. Pulmonary angiography revealed multiple areas of abrupt tapering of the pulmonary arteries, confirming the diagnosis. The patient was switched from bosentan to macitentan and riociguat, and he was referred to our center for PTE.

At admission, his blood pressure was 116/72 mmHg, heart rate was 114 beats per minute, respiratory rate was 18 breaths per minute, and oxygen saturation was 97% on room air. The result of the physical examination was unremarkable. Quantitative hemoglobin S was abnormal at 13.5%, and NT-ProBNP was elevated at 880.0 pg/mL (normal range, 10.0–242.0 pg/mL). The results of the remaining laboratory tests, including coagulation tests, were normal.

**Figure 1:**
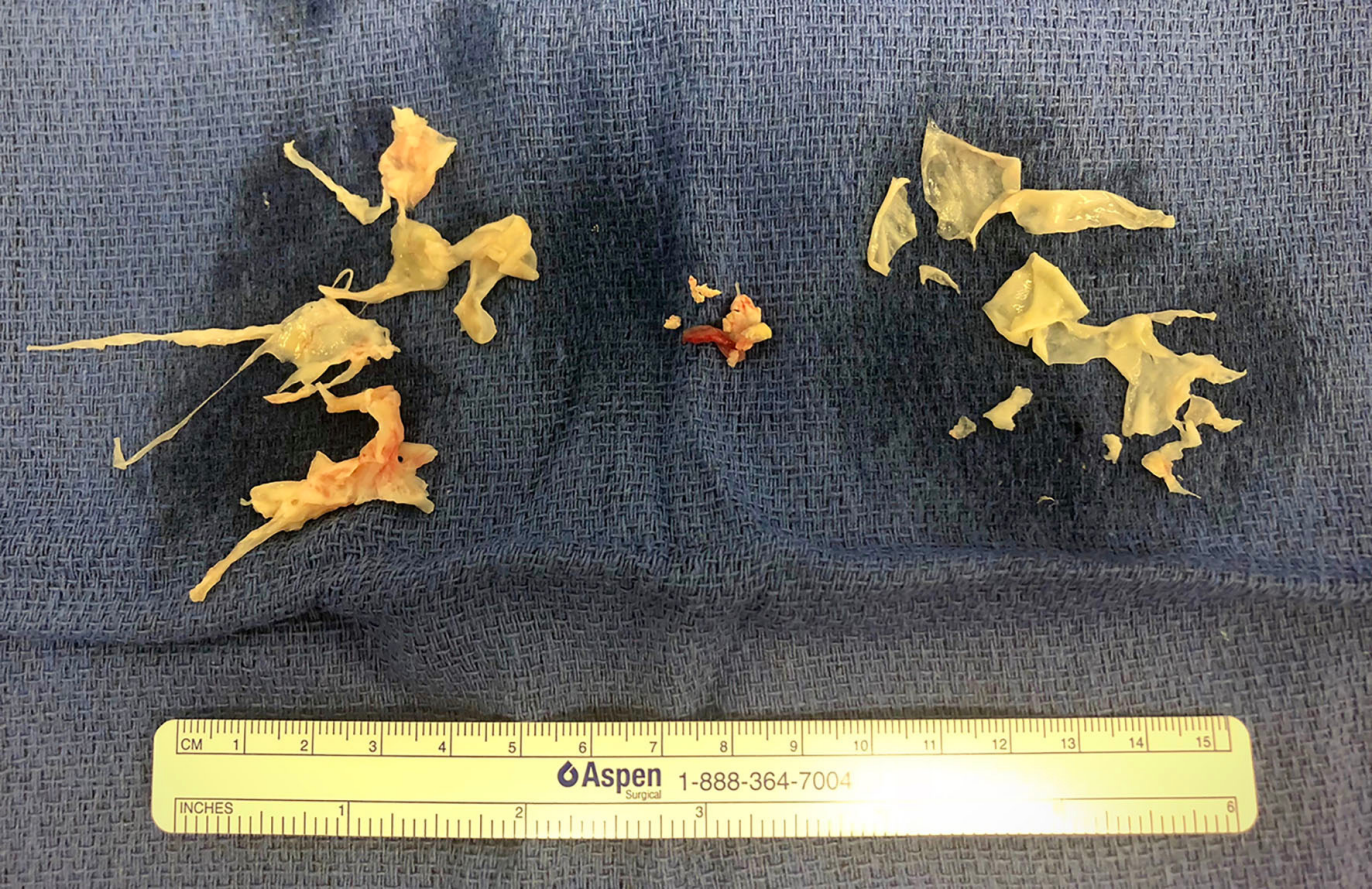
Right atrial thrombus (center) surrounded by pulmonary thromboendarterectomy specimens.

He successfully underwent bilateral PTE and removal of a calcified organized thrombus from the right atrium (Figure 1) without reported intraoperative complications. Post-operative transesophageal echocardiography demonstrated an estimated RV systolic pressure of 32 mmHg plus the RA pressure in the setting of a systolic blood pressure of 110 mmHg, with mildly decreased RV function. Central venous pressure was maintained under 8 mmHg with a furosemide infusion to avoid reperfusion injury. PH medications were discontinued at the time of surgery. Ten days after the procedure, the patient was completely asymptomatic with normal oxygen saturation and was discharged home on long-term warfarin. Four months later, he successfully underwent BMT, after which he had weekly TTEs to monitor for the development of PH (10). As of 6 months following surgery, the patient has remained clinically asymptomatic without echocardiographic evidence of RV hypertension based on tricuspid regurgitant jet and systolic septal position.

## Discussion

To our knowledge, successful PTE in a child with CTEPH and SCD has not been reported in the MEDLINE database to date. Our patient was noted to have unexplained PH on routine screening echocardiography in the setting of a chronic hypercoagulable state and recent RA thrombus associated with a central venous catheter, and his diagnosis was confirmed by lung VQ scan and right heart catheterization. While a recently published case report described a 12-year-old with CTEPH successfully treated with PTE, that patient had severe comorbidities including paraplegia, and he was found to have factor V Leiden and antiphospholipid antibodies during his hypercoagulable workup (11).

CTEPH is a rare and life-threatening condition that can result in progressive right-sided heart failure and death. It occurs as a result of unresolved thrombi obstructing the pulmonary arteries. The following criteria are used to make the diagnosis after 3 months of anticoagulant therapy: (1) mean pulmonary artery pressure >25 mmHg with a pulmonary capillary wedge pressure ≤ 15 mmHg, and (2) at least one (segmental) perfusion defect detected by lung scan, CT angiography, or pulmonary angiography (1, 2, 12).

Despite increasing awareness of it, the disease remains underdiagnosed. Studies suggest an incidence of 0.56–3.2% in adult pulmonary embolism survivors, while incidence in the pediatric population is unknown (3–5). Of note, risk factors for thromboembolism are identified in the majority of children with CTEPH, and approximately one third of patients have a positive family history of thromboembolism or a known hypercoagulable state. Children with lupus anticoagulant and anticardiolipin antibodies are at the highest risk of the disease (9). Other risk factors include splenectomy, infected ventriculo-atrial shunts, thyroid replacement therapy, history of malignancy, chronic inflammatory conditions, and indwelling catheters (13).

Our patient did not have a history of an acute pulmonary embolism. Nevertheless, he had several risk factors for CTEPH. Specifically, he had a history of homozygous SCD, which is recognized as a chronic hypercoagulable state with an increased risk of thromboembolic events and PH (14–17). He also had an indwelling catheter for monthly exchange transfusions and history of catheter-related RA thrombus. Homozygous SCD additionally confers a significant risk of autosplenectomy, for which the patient did not undergo sonographic assessment at our institution (18).

Because there are no pathognomonic signs or symptoms for CTEPH, the diagnosis is often delayed or missed. Patients may present with exertional dyspnea, exercise intolerance, and non-specific abnormalities on physical examination. As the disease progresses, there is a high risk of developing right heart failure. While the natural history of acute pulmonary embolism is near-complete resolution of emboli within 3–6 months, the persistence of any signs or symptoms after this duration of antithrombotic therapy warrants further investigation.

Diagnostic workup begins with chest radiography, pulmonary function studies, an ECG, and an echocardiogram. If CTEPH is suspected, a lung VQ scan should assess for subsegmental or larger unmatched perfusion defects. Given its high sensitivity, a normal lung VQ scan can effectively rule out the disease, while an abnormal test result prompts further evaluation with right heart catheterization, catheter-based pulmonary angiography, CT pulmonary angiography, or MRI (1, 19, 20).

The first step in management is anticoagulant therapy. Our patient was initially on subcutaneous low molecular weight heparin, and he was later transitioned to an oral anticoagulant. Once the diagnosis was established, he was also started on targeted PH therapy, including macitentan, an endothelin receptor antagonist, and riociguat, a soluble guanylate cyclase stimulator, while awaiting definitive surgery. Riociguat was chosen because it has been shown to improve exercise capacity and pulmonary vascular resistance in patients with CTEPH, and because it is safe and well-tolerated in patients with SCD (21, 22).

PTE is the treatment of choice for operable patients, and its success has been demonstrated in children (9, 23). To be considered operable, a patient must have sufficient surgically accessible thromboembolic material without extensive distal disease (24). Patients with SCD may have additional risks of the PTE, given the need for prolonged cardiopulmonary bypass, deep hypothermia, and intervals of circulatory arrest, factors that increase the likelihood of sickling (25). Balloon pulmonary angioplasty is an emerging option for inoperable CTEPH or patients with recurrent or persistent PH after PTE; however, this approach is rarely used in children and long-term results are lacking (26). In our case, a multidisciplinary team including pulmonary hypertension, cardiology, hematology, critical care, and cardiothoracic surgery specialists reviewed the patient's clinical data and elected to proceed with surgery, which the patient underwent without complication. Given the success of this case, it is important to consider CTEPH in any children with unexplained PH, particularly when risk factors are present.

## Conclusions

We describe a rare case of CTEPH in a child with SCD and an indwelling catheter who was found to have unexplained PH. CTEPH is a rare and life-threatening disease. Unlike other forms of PH, it is potentially curable with PTE. For this reason, early recognition and treatment are critical. Practitioners should consider this diagnosis in patients with unexplained PH, particularly in patients with risk factors, including but not limited to those with a hypercoagulable state, a history of thromboembolism, or an indwelling catheter.

## Ethics Statement

Parental informed consent was obtained for the publication of this case report.

## Author Contributions

RS was the consulting cardiology fellow for the patient. GV was the referring cardiologist who diagnosed the patient and he contributed references and revisions to the manuscript. KT performed the patient's thromboendarterectomy and he contributed revisions to the manuscript. ER was the precepting attending for RS and she contributed references and revisions to the manuscript. All authors approved the final version.

## Conflict of Interest

The authors declare that the research was conducted in the absence of any commercial or financial relationships that could be construed as a potential conflict of interest.

## Abbreviations

BMT: bone marrow transplant
CTEPH: chronic thromboembolic pulmonary hypertension
RA: right atrial
RV: right ventricular
PH: pulmonary hypertension
PTE: pulmonary thromboendarterectomy
SCD: sickle cell disease
TTE: transthoracic echocardiogram
VQ: ventilation-perfusion.

## References

[1] Mahmud E Madani MM Kim NH Poch D Ang L Behnamfar O. Chronic thromboembolic pulmonary hypertension. J Am Coll Cardiol. (2018) 71:2468–86. 10.1016/j.jacc.2018.04.009 29793636

[2] Lang IM Pesavento R Bonderman D Yuan JX-J. Risk factors and basic mechanisms of chronic thromboembolic pulmonary hypertension: a current understanding. Eur Respir J. (2013) 41:462–8. 10.1183/09031936.00049312 22700839

[3] Ende-Verhaar YM Cannegieter SC Vonk Noordegraaf A Delcroix M Pruszczyk P Mairuhu ATA. Incidence of chronic thromboembolic pulmonary hypertension after acute pulmonary embolism: a contemporary view of the published literature. Eur Respir J. (2017) 49:1601792. 10.1183/13993003.01792-2016 28232411

[4] Pengo V Lensing AWA Prins MH Marchiori A Davidson BL Tiozzo F. Incidence of chronic thromboembolic pulmonary hypertension after pulmonary embolism. N Engl J Med. (2004) 350:2257–64. 10.1056/NEJMoa032274 15163775

[5] Gall H Hoeper MM Richter MJ Cacheris W Hinzmann B Mayer E. An epidemiological analysis of the burden of chronic thromboembolic pulmonary hypertension in the USA, Europe and Japan. Eur Respir Rev. (2017) 26:160121. 10.1183/16000617.0121-2016 28356407 PMC9488926

[6] Madani MM Auger WR Pretorius V Sakakibara N Kerr KM Kim NH. Pulmonary endarterectomy: recent changes in a single institution's experience of more than 2,700 patients. Ann Thor Surg. (2012) 94:97–103. 10.1016/j.athoracsur.2012.04.004 22626752

[7] Klepetko W Mayer E Sandoval J Trulock EP Vachiery J-L Dartevelle P. Interventional and surgical modalities of treatment for pulmonary arterial hypertension. J Am Coll Cardiol. (2004) 43:S73–80. 10.1016/j.jacc.2004.02.039 15194182

[8] Ishida K Masuda M Tanabe N Matsumiya G Tatsumi K Nakajima N. Long-term outcome after pulmonary endarterectomy for chronic thromboembolic pulmonary hypertension. J Thor Cardiovasc Surg. (2012) 144:321–6. 10.1016/j.jtcvs.2011.09.004 21992851

[9] Madani MM Wittine LM Auger WR Fedullo PF Kerr KM Kim NH. Chronic thromboembolic pulmonary hypertension in pediatric patients. J Thor Cardiovasc Surg. (2011) 141:624–30. 10.1016/j.jtcvs.2010.07.010 20800245

[10] Levy M Moshous D Szezepanski I Galmiche L Castelle M Lesage F. Pulmonary hypertension after bone marrow transplantation in children. Eur Respir J. (2019) 54:1900612. 10.1183/13993003.00612-2019 31649064

[11] Verbelen T Cools B Fejzic Z Van Den Eynde R Maleux G Delcroix M. Pulmonary endarterectomy in a 12-year-old boy with multiple comorbidities. Pulm Circ. (2019) 9:204589401988624. 10.1177/2045894019886249 32284848 PMC7119433

[12] Moser KM Auger WR Fedullo PF. Chronic major-vessel thromboembolic pulmonary hypertension. Circulation. (1990) 81:1735–43. 10.1161/01.CIR.81.6.1735 2188751

[13] Jenkins D Mayer E Screaton N Madani M. State-of-the-art chronic thromboembolic pulmonary hypertension diagnosis and management. Eur Respir Rev. (2012) 21:32–9. 10.1183/09059180.00009211 22379172 PMC9487476

[14] Anthi A Machado RF Jison ML Taveira-DaSilva AM Rubin LJ Hunter L. Hemodynamic and functional assessment of patients with sickle cell disease and pulmonary hypertension. Am J Respir Crit Care Med. (2007) 175:1272–9. 10.1164/rccm.200610-1498OC 17379852 PMC2176091

[15] Gordeuk VR Castro OL Machado RF. Pathophysiology and treatment of pulmonary hypertension in sickle cell disease. Blood. (2016) 127:820–8. 10.1182/blood-2015-08-618561 26758918 PMC4760088

[16] Lim MY Ataga KI Key NS. Hemostatic abnormalities in sickle cell disease: Curr Opin Hematol. (2013). 20:472–7. 10.1097/MOH.0b013e328363442f 23817169

[17] Parent F Lionnet F Habibi A Adnot S O'Callaghan DS Galacteros F. A hemodynamic study of pulmonary hypertension in sickle cell disease. N Engl J Med. (2011) 365:44–53. 10.1056/NEJMoa1005565 21732836

[18] Jais X. Splenectomy and chronic thromboembolic pulmonary hypertension. Thorax. (2005) 60:1031–4. 10.1136/thx.2004.038083 16085731 PMC1747270

[19] Lang IM Madani M. Update on chronic thromboembolic pulmonary hypertension. Circulation. (2014) 130:508–18. 10.1161/CIRCULATIONAHA.114.009309 25092279

[20] Lammers AE Apitz C Zartner P Hager A Dubowy K-O Hansmann G. Diagnostics, monitoring and outpatient care in children with suspected pulmonary hypertension/paediatric pulmonary hypertensive vascular disease. Expert consensus statement on the diagnosis treatment of paediatric pulmonary hypertension. The European Paediatric Pulmonary Vascular Disease Network, endorsed by ISHLT DGPK. Heart. (2016). 102(Suppl. 2):ii1–13. 10.1136/heartjnl-2015-307792 27053692

[21] Ghofrani H-A D'Armini AM Grimminger F Hoeper MM Jansa P Kim NH. Riociguat for the treatment of chronic thromboembolic pulmonary hypertension. N Engl J Med. (2013) 369:319–29. 10.1056/NEJMoa1209657 23883377

[22] Weir NA Conrey A Lewis D Mehari A. Riociguat use in sickle cell related chronic thromboembolic pulmonary hypertension: a case series. Pulm Circ. (2018) 8:204589401879180. 10.1177/2045894018791802 30033820 PMC6083805

[23] Kumbasar U Aypar E Karagöz T Demircin M Dogan R. Pulmonary thromboendarterectomy in pediatric patients: report of three cases. Turk J Pediatr. (2018) 60:604–7. 10.24953/turkjped.2018.05.023 30968628

[24] Pepke-Zaba J Delcroix M Lang I Mayer E Jansa P Ambroz D. Chronic thromboembolic pulmonary hypertension (CTEPH): results from an international prospective registry. Circulation. (2011) 124:1973–81. 10.1161/CIRCULATIONAHA.110.015008 21969018

[25] Yung GL Channick RN Fedullo PF Auger WR Kerr KM Jamieson SW. Successful pulmonary thromboendarterectomy in two patients with sickle cell disease. Am J Respir Crit Care Med. (1998) 157:1690–3. 10.1164/ajrccm.157.5.9710032 9603156

[26] Lang I Meyer BC Ogo T Matsubara H Kurzyna M Ghofrani H-A. Balloon pulmonary angioplasty in chronic thromboembolic pulmonary hypertension. Eur Respir Rev. (2017) 26:160119. 10.1183/16000617.0119-2016 28356406 PMC9489135

